# A Survival Analysis of Acute Myeloid Leukemia Patients Treated With Intensive Chemotherapy: A Single Center Experience

**DOI:** 10.7759/cureus.43794

**Published:** 2023-08-20

**Authors:** Laura Jimbu, Madalina Valeanu, Adrian P Trifa, Oana Mesaros, Anca Bojan, Delia Dima, Andrada Parvu, Ioana C Rus, Ciprian Tomuleasa, Tunde Torok, Laura Urian, Anca Vasilache, Mihnea Zdrenghea

**Affiliations:** 1 Hematology, ‘Ion Chiricuta’ Oncology Institute, Cluj-Napoca, ROU; 2 Hematology, Iuliu Hatieganu University of Medicine and Pharmacy, Cluj-Napoca, ROU; 3 Statistics, Iuliu Hatieganu University of Medicine and Pharmacy, Cluj-Napoca, ROU; 4 Genetics, Iuliu Hatieganu University of Medicine and Pharmacy, Cluj Napoca, ROU; 5 Genetics, 'Ion Chiricuta' Oncology Institute, Cluj-Napoca, ROU; 6 Hematology, 'Ion Chiricuta' Oncology Institute, Cluj, ROU; 7 Hematology, 'Ion Chiricuta' Oncology Institute, Cluj Napoca, ROU; 8 Hematology, 'Ion Chiricuta' Oncology Institute, Cluj-Napoca, ROU

**Keywords:** infections, early mortality, fit patients, intensive chemotherapy, acute myeloid leukemia (aml)

## Abstract

Introduction: Acute myeloid leukaemia (AML) is a haematological disease associated with a dismal prognosis, despite major progress made in recent years in terms of antileukemic agents and supportive care.

Methods: We investigated the results of the intensive treatment of 133 fit AML patients (*de novo* and secondary) from a referral cancer centre in Romania, treated between January 2015 and December 2021.

Results: We included 79 male and 54 female patients with a median age of 53 years (range 18-70). Molecular biology analysis was available for 82.7% of patients, whereas karyotype analysis was only available for 33% of patients. The median overall survival (OS) was 8.7 months, and the disease-free survival rate was 26.3% at a median follow-up of 33.7 months. The complete remission (CR) rate after induction was 48.9% for all patients and 61.9% for patients who were assessable (excluding patients who died before being assessed for response). Twelve patients underwent allogeneic bone marrow transplantation (BMT), with the median OS not reached. Early mortality (EM), defined as death during the first 30 days after admission, was 17.3%, with the main cause of death being septic shock (78.3%). Elderly patients (≥60 years of age) had a lower OS, more primary refractory disease, and higher rates of early mortality.

Conclusion: Complete remission rates and OS in our cohort were lower than in other reports. Early mortality was unexpectedly high, mainly due to infections, which were the main causes of death in our cohort.

## Introduction

Acute myeloid leukaemia (AML) is a haematological malignancy characterized by the proliferation of abnormal myeloid precursors, termed blasts, leading to bone marrow failure. In 1973, Yates et al. proved that the combination of daunorubicin administered over three days and cytarabine administered for a week (‘3+7’) led to a significant complete remission (CR) and overall survival (OS) rate, and, later, this regimen was established as the gold standard for treating fit AML patients [1]. In 1981, another study confirmed the superiority of ‘3+7’ compared to other regimens and to the shorter version of ‘5+2’ [2]. Based on the progress made in understanding the biology of AML and in developing new supportive therapies, the long-term survival rate of patients aged below 60 years increased from 13% in the 1970s to 55% in 2010, according to a study conducted at the MD Anderson Cancer Centre [3]. Although in most settings, ‘3+7’ remains the backbone of the first-line treatment for fit, young patients, others switched to different intensive regimens such as FLAG-IDA (fludarabine 30 mg/m^2^ + cytarabine 2 g/m^2^ + idarubicin 8 mg/m^2^ + granulocyte colony-stimulating factor [G-CSF] 5 mcg/kg) [3,4]. Better knowledge of AML biology has been translated into new therapies such as FLT3 inhibitors, IDH1/IDH2 inhibitors, bi-specific T-cell engagers (e.g., flotetuzumab), anti-CD33 antibody-drug conjugates, hypomethylating agents, and BCL2 inhibitors. In 2022, the European Leukaemia Net (ELN) published a new set of recommendations that refined the diagnosis and treatment of AML, mostly based on karyotyping and molecular analysis [5].

Although clinical trials have demonstrated very good treatment results, real-world data are also important in providing unbiased information regarding CR and OS in AML patients. A Nigerian study reported a very low OS, mostly because of limited supportive care and scarce infrastructure for bone marrow transplantation (BMT). Also, even though clinicians have administered reduced doses of chemotherapy, early mortality (EM) in patients with AML is still very high, at about 70%. Furthermore, Nigeria lacks facilities that perform cytogenetic and molecular testing [6]. In a study from a referral centre in Egypt, the CR rate was about 65%, which is similar to most reports, and molecular testing (13%) and karyotyping (27%) were rarely used [7]. In contrast, a real-world study from the Czech Republic reported excellent results in young patients with AML with an EM of only 3.9%, a CR rate of 70.1%, and a two-year and five-year OS of 54% and 44%, respectively [8]. A single-centre study from Mexico showed that whereas CR rates were good in their cohort, BMT was rarely performed, especially in the adult cohort compared to adolescents, because of a lack of infrastructure as well as social and cultural aspects [9].

## Materials and methods

We performed a retrospective analysis evaluating young/fit patients diagnosed with AML (de novo and secondary) and treated intensively over a period of seven years in a referral cancer centre, the ‘Ion Chiricuta’ Oncology Institute from Cluj-Napoca, Romania. Patients included in this study were diagnosed with AML between January 1, 2015, and December 31, 2021. Patients were excluded from our study if they were diagnosed with biphenotypic leukaemia, acute promyelocytic leukaemia, or blastic plasmacytoid dendritic cell neoplasm, or were only diagnosed in our centre and treated elsewhere. We included all patients diagnosed with AML aged greater than 18 years who were treated with aggressive chemotherapy. We recorded their age, gender, date of arrival in our clinic, haemoglobin level, platelet count, leukocytes, blasts in peripheral blood and bone marrow, ferritin, fibrinogen, lactate dehydrogenase (LDH), erythrocyte sedimentation rate (ESR), molecular biology, karyotype, procalcitonin, treatment and response to treatment, cause of death, date of death/last visit in our clinic, number of cases in which antibiotic prophylaxis was used, and the type of antibiotic administered. The analysis was performed by reviewing patients’ medical and digital records, and the collected data were entered into an Excel spreadsheet. Statistic correlations and Kaplan-Meier survival curves were generated using SPSS Statistics (IBM Corp., Armonk, NY) and GraphPad Prism 9 (GraphPad Software, Inc., La Jolla, CA). Patients were censored on April 29, 2022. Patients lost to follow-up were censored at the last date they were present in our clinic. P-values less than 0.05 were considered statistically significant. The study was performed in accordance with the Helsinki declaration.

## Results

Risk assessment and response criteria were based on the 2022 ELN recommendations [5]. Risk categories were stratified according to the ELN, but where karyotyping was not available, we based our stratification solely on molecular biology. EM was defined as death in the first 30 days after admission to our clinic.

We enrolled 133 patients diagnosed with AML and treated intensively in our cancer centre between January 2015 and December 2021. A diagnosis was made based on a bone marrow cytological examination, or bone marrow biopsy, and flow cytometry of peripheral blood and/or bone marrow. The median patient age was 53 years (range 18-70), with a predominance of the male gender (male/female ratio 1.46:1). Patients’ characteristics are presented in Table 1.

**Table 1:** Characteristics of patients diagnosed with acute myeloid leukaemia and treated intensively. ESR, erythrocyte sedimentation rate; LDH, lactate dehydrogenase; N/A, not available.

Variable		Number of patients	Percentage (%)
Gender	Male	79	59.40
	Female	54	40.60
Age (years)	18-29	9	6.77
	30-39	11	8.27
	40-49	32	24.06
	50-59	41	30.83
	60-69	39	29.32
	70-79	1	0.75
Median	53		
Year of diagnosis	2015	18	13.53
	2016	20	15.04
	2017	16	12.03
	2018	24	18.05
	2019	20	15.04
	2020	14	10.53
	2021	21	15.79
Molecular biology	Yes	110	82.71
	N/A	23	17.29
NPM1	Mutated	35	26.32
	Wild-type	71	53.38
	N/A	27	20.30
FLT3 ITD	Mutated	20	15.04
	Wild-type	82	61.65
	N/A	31	23.31
FLT3 TKD	Mutated	10	7.52
	Wild-type	91	68.42
	N/A	32	24.06
Others	BCR-ABL	1	0.75
	CBFB-MYH11	1	0.75
	AML-ETO	6	4.50
	DEK-CAN	1	0.75
	NUP98-NSD1	1	0.75
Karyotype	Normal	24	18.04
	Abnormal	20	15.04
	N/A	89	66.92
Risk stratification	Favourable	21	15.8
	Intermediate	68	51.1
	Adverse	15	11.3
	N/A	29	21.8
Haemoglobin (g/l)	Min	38	
	Max	132	
	Median	88	
Platelets (10^9^/l)	Min	2	
	Max	286	
	Median	41	
Leukocytes (10^9^/l)	Min	0.19	
	Max	347	
	Median	24.3	
	<40	77	
	≥40 to <100	28	
	≥100	28	
Peripheral blasts (%)	Min		0
	Max		99
	Median		35
Bone marrow blasts (%)	Min		20
	Max		100
	Median		50
Ferritin (ng/ml)	Min	79	
	Max	5364	
	Median	770	
LDH (U/L)	Min	197	
	Max	10677	
	Median	1174	
ESR (mm/h)	Min	2	
	Max	170	
	Median	88	
Fibrinogen (mg/dl)	Min	120	
	Max	1215	
	Median	435	

Of the 133 patients, 110 (82.7%) had been tested by molecular biology, of which 35 (26.3%) were NPM1 positive, 20 (15%) were FLT3 ITD positive, 10 (7.5%) were FLT3 TKD positive, and 10 (7.5%) harboured other mutations. Karyotyping was available in 44 patients (33%), which was abnormal in 20 (15%) patients. Risk assessment showed that 15.8%, 51.1%, and 11.3% of patients had favourable, intermediate, and adverse risks, respectively. Of note, 21.8% of patients could not be assessed due to a lack of molecular biology (either a lack of NPM1, FLT3, ITD/TKD, or both) and/or karyotyping.

**Figure 1:**
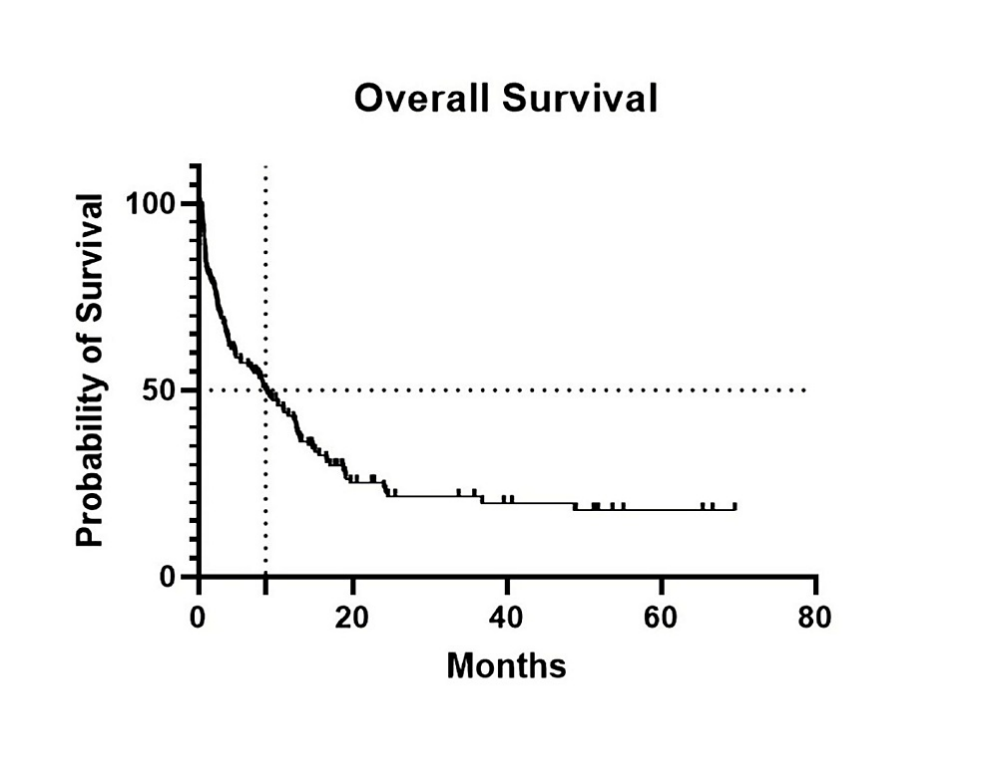
The median OS in patients treated intensively was 8.7 months at a follow-up of 33.7 months.

**Figure 2:**
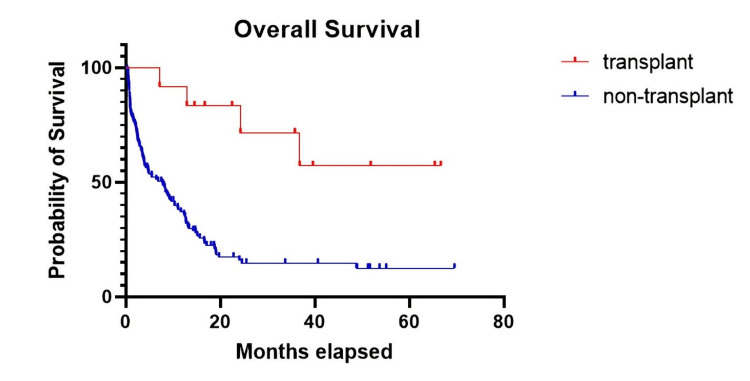
The median OS in patients who underwent transplant was not reached compared to non-transplanted patients where the median OS was 7.7 months at a follow-up of 33.7 months.

At follow-up, of the 133 patients included in the study, 98 (73.7%) were dead and 35 (26.3%) were alive, with a median OS of 8.7 months at a median follow-up of 33.7 months (Figure 1). Only 9% of patients underwent allogeneic BMT (12/133). OS was statistically better in patients who underwent allogeneic BMT compared to non-transplanted patients (p = 0.0004, hazard ratio [HR] = 0.2, Figure 2), with a median OS of 7.7 months in non-transplanted patients compared to transplanted patients, where the median OS was not reached.

As induction therapy, 97 (72.9%) patients were administered the standard ‘3+7’ regimen, 14 (10.5%) were administered ‘3+7’ + etoposide, 11 (8.3%) were administered ‘3+7’ + an FLT3 inhibitor (e.g., midostaurin, quizartinib), three (2.3%) were administered ‘3+7’ + glasdegib, six (4.5%) were administered ‘5+2’ or ‘5+3’, and two (1.6%) were administered other regimens. Of the 133 patients, 28 (21.1%) died during induction, 65 (48.9%) obtained a CR and proceeded to consolidation, and 40 (30%) were refractory to the first course of induction. If we excluded patients who died before the assessment of response, the CR rate increased to 61.9%. Tables 2-3 present the types of induction and consolidation and the respective response rates. Of the patients in CR, 59 (89.4%) proceeded to first consolidation with high-dose cytarabine (HiDAC) at 1.6-3 g/m^2^ every 12 hours on days 1, 3, and 5 or days 1, 2, and 3, and three (4.5%) patients were administered intermediate cytarabine (IDAC 1-1.5 g/m^2^ of cytarabine on the same days as previously stated in the HiDAC regimen), one patient was administered low-dose cytarabine, one was administered FLAG, and one was administered a repeat course of ‘3+7’. In four patients, the consolidation regimen was changed after the first or second course of consolidation due to complications attributable to drug toxicity (e.g., infectious, neurological) or due to temporary cytarabine shortage not allowing for a HiDAC regimen administration.

**Table 2:** Induction treatment and responses. CR, complete response.

	Number of patients	Percentage (%)
Induction		
3+7	97	72.9
3+7+etoposide	14	10.5
3+7+ FLT3 inhibitor	11	8.3
3+7+glasdegib	3	2.3
5+2/5+3	6	4.5
Other	2	1.6
Response to induction		
Death	28	21
CR	65	48.9
Refractory	40	30.1

**Table 3:** Consolidation treatment and response. HiDAC, high-dose cytarabine; CR, complete response; IDAC, intermediate cytarabine.

	Number of patients	Percentage (%)
First consolidation		
HiDAC	59	90.8
IDAC	3	4.6
Low-dose cytarabine	1	1.5
3+7	1	1.5
FLAG	1	1.5
Response to first consolidation		
Death	3	4.6
CR	58	89.2
Relapse	4	6.2
Second consolidation		
HiDAC	47	84
IDAC	6	10.7
FLAG	1	1.7
Low-dose cytarabine	2	3.6
Response to the second consolidation		
Death	2	3.6
CR	52	92.8
Relapse	2	3.6
Third consolidation		
HiDAC	41	80.3
IDAC	4	7.8
FLAG	1	2
3+7	1	2
Low-dose cytarabine	3	5.9
Mitoxantrone + etoposide	1	2
Fourth consolidation		
HiDAC	8	72.7
IDAC	1	9.1
FLAG	1	9.1
Low-dose cytarabine	1	9.1
Response at follow-up after either 3 or 4 consolidations		
Death	2	47
CR	25	49
Relapse	24	4

Of the 65 CR patients proceeding through the three cycles of consolidation, seven died during treatment, six relapsed during consolidation, three were in the course of treatment at follow-up, and 49 were still in CR at the end of the three cycles. Of these, 11 patients received a fourth cycle of consolidation.

At the end of treatment, out of the 133 patients included in our study, 28 died during induction, three died during the first consolidation, and two each died during the second and third consolidation, respectively. None died during the fourth consolidation. Eventually, at follow-up, of the 49 patients completing the first-line treatment, only 25 were still in CR and 24 had relapsed. Of the 25 patients in CR, one died of unknown causes after completing treatment. Overall, 40 patients were refractory to first-line treatment; six relapsed during consolidations, and 24 relapsed after completing consolidation and were eligible for second-line treatment. Four died before proceeding with salvage chemotherapy.

Sixty-six patients underwent second-line treatment: 38 of those were refractory to first-line treatment, and 28 of those relapsed. Ten (15.1%) patients received a second course of induction (‘3+7’ or ‘5+3’), 42 (63.6%) received fludarabine-based regimens, and the other 14 (21.2%) were administered other regimens, either aggressive or low intensity. At follow-up, after reinduction (patients who received the same regimen as their first-line treatment) or second-line treatment, 14 patients were in CR, 11 achieved CR but relapsed later, 20 died during treatment, 20 were refractory, and one was still under treatment. Eleven (8.3%) patients were primary refractory, defined as not achieving CR after two cycles of intensive chemotherapy [5]. Thirty-one patients received third-line treatment. Of these patients, only two achieved CR, nine died during treatment, 16 were refractory, one was not assessable, and three achieved CR but relapsed later. Table 4 presents the second- and third-line treatments and the responses to them. Eleven patients received fourth-line treatment, of which five died during treatment and none achieved CR.

**Table 4:** Second-line treatment and subsequent lines and responses. HiDAC, high-dose cytarabine; CR, complete response; N/A, not available.

Second line	Number of patients	Percentage (%)
FLAG	36	54.6
3+7/5+3	10	15.2
HiDAC	7	10.6
FLAG + etoposide/idarubicin	6	9.1
Azacitidine	3	4.5
Others	4	6
Response to the second line		
CR	14	21.2
Death	20	30.3
Relapse	11	16.7
Refractory	20	30.3
N/A	1	1.5
Response to the third line		
CR	2	6.5
Death	9	29
Relapse	3	9.7
Refractory	16	51.6
N/A	1	3.2

**Figure 3:**
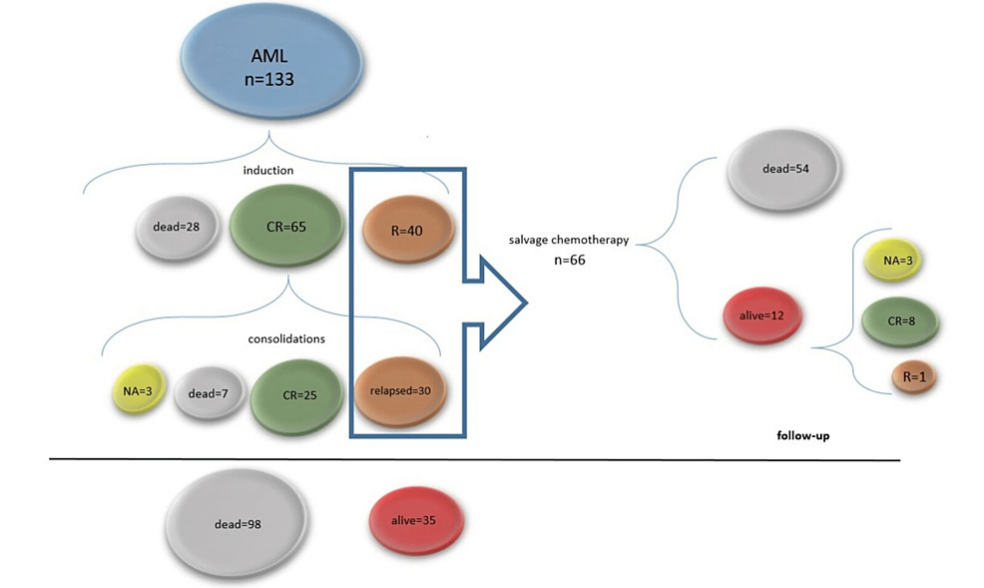
The response of 133 AML patients to treatment. During induction, 28 patients died, 65 achieved CR, and 40 were refractory (R). All patients who achieved CR proceeded to consolidation. Of these, at follow-up, three were still undergoing treatment, seven died, 25 were in CR and 30 were relapsed (either during or after consolidation). From the 25 patients in CR, one died of unknown causes. Of the 70 eligible patients, 66 patients received salvage treatment, and four died before second-line treatment. At follow-up, of these patients, 54 were dead and 12 were still alive, eight were in CR, three were not assessable, and one was refractory to treatment. At follow-up, 98 patients were dead and 35 were alive, of which 28 were in CR, one was refractory to treatment, and 6 were not yet assessed for a response.

Figure 3 briefly summarizes the response of the 133 AML patients to treatment. Three patients received fifth-line treatment, after which two were still refractory and one was not assessable. One patient received two other courses of treatment but received no response.

Patients who did not achieve CR after the first induction, relapsed during or after consolidation, or were classified as having an adverse risk according to the ELN classification were referred to a bone marrow transplantation facility for allogeneic stem cell transplantation.

In our cohort, the EM rate was 17.3%. In 18 (78.3%) patients, the cause of death was septic shock. Table 5 presents the cause of death in patients with EM, and Table 6 presents the bacteria or fungi associated with septic shock.

**Table 5:** Cause of death in acute myeloid leukaemia patients.

Cause of death	Number of patients
Septic shock	18
Stroke	2
Cardiovascular complications	1
Bleeding	1
Multiple organ dysfunction syndrome	2

**Table 6:** Pathogens implicated in septic shock. N/A, not available.

Pathogens	Number of patients
Bacteria involved in septic shock	
Acinetobacter baumannii	3
Escherichia coli	2
Klebsiella pneumonia	2
Clostridium difficile	1
Enterococcus faecalis	1
Methicillin-resistant Staphylococcus epidermidis	2
Methicillin-resistant Staphylococcus xylosus	2
N/A	7
Detected fungi	
Candida albicans	1
Aspergillus	1
Stenotophomonas maltophilia	2
Candida famata	1
Candida parapsilosis	1

Currently, we only use antibiotic prophylaxis seldom. However, they received antifungal prophylaxis, mainly with fluconazole. In our study, 26 (19.5%) patients received antibiotic prophylaxis, but in most cases, these drugs were not administered on a regular basis but rather only during one or two cycles of chemotherapy, and sometimes different antibiotics were administered between cycles. Table 7 presents the type of antibiotic used for prophylaxis.

**Table 7:** Types of prophylactic antibiotics used.

Drug	Number of patients
Fluoroquinolone	8
Cephalosporine	6
Trimethoprim/sulfamethoxazole	7
Amoxicillin and clavulanic acid	1
Fluoroquinolone + cephalosporine	4

As a standard procedure, after CR was obtained, usually during consolidations, patients received G-CSF until the number of neutrophils normalized. Unfortunately, we do not have a standardized approach to antibiotic usage in our clinic. In the past, fluoroquinolones were used for prophylaxis in neutropenic patients, and the combination of a fluoroquinolone and a third-generation cephalosporin was commonly used for the treatment of febrile neutropenia. Later, this approach was abandoned due to the high number of patients who developed Clostridium difficile infection (CDI). Thus, imipenem-cilastatin alone or in combination with vancomycin was considered a more appropriate and safer option for our patients with febrile neutropenia. However, prophylaxis was abandoned or performed according to the treating physician's preference but in a non-standardized manner.

**Figure 4:**
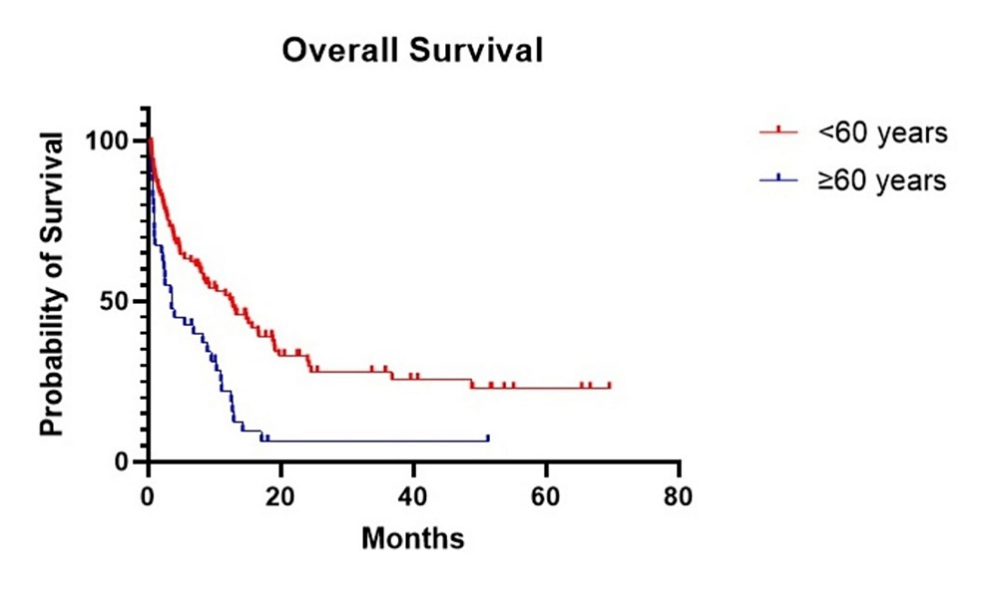
The OS of patients aged <60 years was 12.7 months whereas in patients aged ≥60 years, the median OS was 3.47 months.

We divided patients into two groups based on their age: <60 years and ≥60 years. Patients under 60 years had a prolonged OS compared to those ≥60 years (p<0.001, HR=0.44). The median OS of patients aged <60 years was 12.7 months, whereas the median OS for patients aged ≥60 years was 3.47 months (Figure 4).

We also evaluated gender (p=0.43), haemoglobin (p=0.23), ferritin (p=0.93), number of leukocytes (p=0.63), platelets (p=0.38) number of blasts in bone marrow (p=0.49), number of blasts in peripheral blood (p=0.61), ESR (p=0.1), LDH (p=0.92), procalcitonin (p=0.99), and fibrinogen (p=0.056), and correlated them with the CR and OS, although no statistical significance was found. Adverse karyotyping was not associated with lower CR rates or OS. We also tried to evaluate whether ‘3+7’ + etoposide improved survival compared to ‘3+7’, but there was no statistical significance in the OS survival (p=0.35). As previously mentioned, the most common cause of death in our cohort was septic shock. In our study, we correlated EM with different parameters, but only age (<60 vs. ≥60 years) was statistically significant, suggesting that patients aged ≥60 years were more prone to EM (p=0.004, HR=0.53). Also, primary refractory disease was correlated with age (p=0.048, OR=3.1, 95% CI 0.1-10.85) and with adverse risk according to the ELN classification (p=0.042).

## Discussion

Our results were surprisingly dismal. The EM of 17.3% was unexpectedly high in our cohort, and it was even higher in our elderly patients (data not shown). The main cause of death was septic shock. We do not have a single standardized approach for febrile neutropenia in our department, with some practitioners preferring an ‘escalation’ versus a ‘de-escalation’ approach [10]. The most frequent antibiotic used is imipinem-cilastatin as a single agent or in combination with vancomycin. Prophylaxis is not a standard procedure in our centre. To improve our results, we might consider using antibiotic prophylaxis with quinolones, which was previously a quasi-standard procedure in our facility but was abandoned a few years ago due to the high incidence of CDI. It is now well known that fluoroquinolones are associated with significant gut dysbiosis [11,12]. Reported EM rates vary depending on the cohort studied. A review from Hahn et al. reported 26.7% EM in the Surveillance, Epidemiology, and End Results (SEER) cohort compared to 12.2% in the Southwest Oncology Group (SWOG) cohort, but, unlike in our study, they included patients that were fit and unfit [13]. Another review from the SEER database showed that the EM decreased from 18.7% in the 1970s to 5.8% in patients diagnosed from 2008 to 2010, who were treated with intensive chemotherapy [14]. A study from Turkey reported an EM rate, defined as death in the first 15 days after initiating therapy, of 10.67% in patients <60 years old [15].

Of the 133 patients included in our study, 79 were male and 54 were female, confirming the same gender bias reported by most previous studies [16,17].

We found no statistically significant differences in gender regarding the OS or CR rates, but previous reports suggested gender might have an influence. Hellesøy et al. showed that concurrent FLT3 ITD, NPM1, and DNMT3A were more frequent in females, whereas RUNX1, SRSF2, U2AF1, ASXL1, and EZH2 were more frequent in male patients. However, certain mutations, such as ZNF711, were present only in women, suggesting that gender might influence prognosis [18]. Wiernik et al. reported that females with non-promyelocytic AML had a better OS [19], whereas Acharya et al. demonstrated that male gender was associated with a lower OS [20].

In our study, age ≥60 years correlated with a lower OS, increased EM, and primary refractory disease. Other studies suggest the importance of age regarding the EM, but with different cut-off values [21-24]. It was suggested that age and refractoriness were correlated mainly because elderly patients have complex karyotypes, secondary AML, or treatment-related AML [25].

Although our study did not demonstrate that leukocytosis has a prognostic impact on the OS, CR rate, or event-free survival, some studies have reported otherwise [26,27]. Another study showed that low levels of platelets and low haemoglobin were associated with a lower OS [28].

In our study, the median OS was 8.7 months, with a long-term survival of 26.3% and a median follow-up of 33.7 months. In other countries, such as the USA, the five-year survival rate improved from 13% in the 1970s to 49% in 2010, probably due to early access to BMT, better supportive care, and more clinical trials [3]. A study from Sweden reported a CR rate of 65% in patients treated intensively with de novo AML [29], whereas a study from Brazil reported an OS of 17% at five years [30]. A retrospective study of 9,380 fit patients treated intensively showed that the median OS increased from six months to 23 months in patients treated intensively (1973-1977 vs. 2008-2010) [14].

## Conclusions

This retrospective study assessed the response to treatment in patients with AML treated with intensive chemotherapy. The median OS was 8.7 months, and the disease-free survival rate was 26.3% at a median follow-up of 33.7 months. These results are lower than in other reports. EM was unexpectedly high, mainly due to infections, which were the main cause of death in our cohort. Antibiotic prophylaxis might be an effective way to prevent septic shock and, thus, death.
